# Supplementary material for: A lexical approach for identifying behavioural action sequences
Source: PLoS Comput Biol. 2022 Jan 10;18(1):e1009672. doi: 10.1371/journal.pcbi.1009672 (PMC8782473; doi:10.1371/journal.pcbi.1009672)
Supplement: S1 Table — The 25 motifs that deviate most from Markovianity are shown (as measured by a p-value with Markovianity as the null hypothesis, see main text). (PDF) [file pcbi.1009672.s006.pdf]

S1 Table: Motifs discovered in exploratory data. The 25 motifs that deviate most from Markovianity are shown (as measured by a p-value with Markovianity as the null hypothesis, see main text).

**Motif**	$-\log_{10} p$	**Observed**	**Expected**
ffffffffff	inf	1366	387
ffffffffffffff	inf	510	50
fffffff	208.01	3234	1797
ffff	42.33	9544	8327
FFFFFFF	28.23	311	153
fffffftf	27.64	497	290
fftfffff	25.07	495	297
fftfff	22.72	1125	824
fftff	21.12	1745	1377
fftf	18.5	2724	2289
ftf	13.96	4337	3859
TfT	11.12	722	554
FFFF	7.94	1428	1224
TfTf	7.28	346	254
tttt	6.7	256	181
TTTT	5.06	160	110
bb	3.87	924	1044
bbbb	3.21	115	82
FbFb	2.19	99	74
Fb	1.79	1300	1388
Tfff	1.74	1690	1788
bbFb	1.72	72	54
bFbF	1.72	100	78
bF	1.66	1374	1460
bFb	1.57	242	209
