# Supplementary material for: A lexical approach for identifying behavioural action sequences
Source: PLoS Comput Biol. 2022 Jan 10;18(1):e1009672. doi: 10.1371/journal.pcbi.1009672 (PMC8782473; doi:10.1371/journal.pcbi.1009672)
Supplement: S2 Table — The 25 motifs that deviate most from Markovianity are shown (as measured by a p-value with Markovianity as the null hypothesis, see main text). (PDF) [file pcbi.1009672.s007.pdf]

S2 Table: Motifs discovered in the data from Marques et al. The 25 motifs that deviate most from Markovianity are shown (as measured by a p-value with Markovianity as the null hypothesis, see main text).

**Motif**	$-\log_{10} p$	**Observed**	**Expected**
TTTTTTT	142.48	580	161
TTTTTTTT	142.23	378	71
fffffff	132.95	883	338
ffffffff	130.9	546	155
TTTTTT	128.43	915	364
ffffff	127.1	1483	741
fTf	119.55	1896	1057
fffff	105.39	2569	1620
TTTTT	102.33	1515	826
ffTf	96.1	1009	483
ffTff	72.61	540	221
tttttt	64.91	475	192
ffff	64.26	4586	3544
ffffTf	59.68	306	101
TTTT	57.99	2604	1870
tttttttt	56.51	148	28
TfTf	55.98	355	133
FFFFFF	53.79	385	154
fffTfff	51.81	184	46
FfF	50.8	1447	948
FFFFFFF	47.97	201	57
TffTf	47.27	206	61
ffTfffff	42.53	111	21
ttff	42.3	881	1352
tttt	42.11	1853	1326
fFf	42.09	2217	1637
ffffTfff	41.28	110	21
