# Supplementary material for: A lexical approach for identifying behavioural action sequences
Source: PLoS Comput Biol. 2022 Jan 10;18(1):e1009672. doi: 10.1371/journal.pcbi.1009672 (PMC8782473; doi:10.1371/journal.pcbi.1009672)
Supplement: S3 Table — The 25 motifs that deviate most from Markovianity are shown (as measured by a p-value with Markovianity as the null hypothesis, see main text). (PDF) [file pcbi.1009672.s008.pdf]

S3 Table: Motifs discovered in the data from chemotactic fish in the aversive environment. The 25 motifs that deviate most from Markovianity are shown (as measured by a p-value with Markovianity as the null hypothesis, see main text).

**Motif**	$-\log_{10} p$	**Observed**	**Expected**
ffffffffffffff	inf	497	33
ffffffffffff	inf	751	93
ffffffffffffffff	inf	334	12
ffffffff	278.21	1860	719
ffffff	155.38	3266	1990
FFFFFFF	71.86	653	294
ffff	44.26	6533	5506
FFFFFFFFFFFFFF	43.87	59	4
ffftffff	17.45	290	166
ffffftff	16.56	285	165
FFFF	14.89	2167	1821
ffftff	14.89	641	460
fftff	10.83	960	767
TfT	8.42	456	342
fTfT	6.54	184	123
bbbb	6.45	316	234
tttt	6.09	162	107
bFFFFFFF	5.94	87	49
FFFFFFFb	5.67	83	47
TTTT	5.3	139	92
ftff	4.87	1448	1290
FFFbFFFF	4.79	56	30
bFb	4.18	405	330
bbFb	3.81	148	107
Obbb	3.68	106	72
