# Supplementary material for: A lexical approach for identifying behavioural action sequences
Source: PLoS Comput Biol. 2022 Jan 10;18(1):e1009672. doi: 10.1371/journal.pcbi.1009672 (PMC8782473; doi:10.1371/journal.pcbi.1009672)
Supplement: Attachment — Submitted filename: response_letter_final.docx [file pcbi.1009672.s012.docx]

Dear Editors,

We thank the reviewers for considering our work. The response to the reviewers’ comments are attached. A few additional minor edits were made during our revisions, which are noted further below.

A PDF of the revised manuscript with the changes highlighted is attached. The changes corresponding to the comments by Reviewer #1 are highlighted in **blue**, Reviewer #2 in **yellow** and additional changes in **green**.

Reviewer #1:

"A lexical approach for identifying behavioral action sequences" by Reddy et al. draws on an analogy between behaviour and language to develop a new model of behaviour sequences. The model is related to models first developed in bioinformatics but has been extended to deal with differences between language and DNA sequence data (which are intrinsically discrete) and behavioural data where discretisation is noisy. The authors show they can fit their model with an EM algorithm on simulated data, zebrafish behaviour data, and on simulated glider motion. They identify rare but conserved sequences with interesting properties. For example, they find that slow and fast bouts occur in separate motifs (as opposed to being interspersed with each other). The paper is engaging and clearly written.

We thank the reviewer for the very useful feedback to improve our manuscript.

I have only two main (interrelated) concerns. The first is that the model is motivated using a contrast with recent methods for behaviour classification, arguing that these methods don't capture behavioural sequences. As currently presented the introduction elides the long history in ethology of trying to capture and analyse behavioural sequences. To give just a few examples that explicitly mention the connection to language:

-The problem of serial order in behavior KS Lashley - 1951

-Grammar of a movement sequence in inbred mice JC Fentress, FP Stilwell
Nature, 1973

-Natural syntax rules control action sequence of rats KC Berridge, JC Fentress, H Parr
Behavioural brain research, 1987

-Linguistic analogies and behavior: The finite-state behavioral grammar of food-hoarding in hamsters CH Jones, JPJ Pinel - Behavioural brain research, 1990

-A syntax of hoverfly flight prototypes BRH Geurten, R Kern, E Braun, M Egelhaaf
Journal of Experimental Biology 2010

-Changes in Postural Syntax Characterize Sensory Modulation and Natural Variation of C. elegans Locomotion RF Schwarz, R Branicky, LJ Grundy, WR Schafer, AEX Brown
PLOS Comp Biol 2015

-Grammars of action in human behavior and evolution D Stout, T Chaminade, A Thomik, J Apel, A Faisal bioRxiv 2018

-Drosophila melanogaster grooming possesses syntax with distinct rules at different temporal scales JM Mueller, P Ravbar , JH Simpson, JM Carlson PLOS Comp Biol 2019

I realise there is a massive literature here and I'm not suggesting the authors need to write a history of ethology in their introduction, nor am I suggesting this takes away from their new model which is an exciting contribution. Instead, I think it would be useful to frame the introduction slightly differently around the idea that ethology has long been concerned both with identifying behaviours and with understanding their sequences. Much of the recent work in computational ethology (but not all) has focussed on the first problem. The contribution of the current paper is a new model to solve the second problem. But rather than being the first approach to the second problem, it builds on existing approaches. This should be reflected especially in the second paragraph of the introduction which currently has no citations at all.

We thank the reviewer for the detailed suggestions and references. We have added a paragraph in the introduction discussing the dichotomy between segmenting bouts or modeling the dynamics and segmenting action sequences in more detail (see lines 22-33). We highlight that our contribution is to build a generative, probabilistic framework for segmenting action sequences, which can be extended in different directions in future work. The references suggested by the reviewer are now cited (see highlighted changes in references) at appropriate locations.

My second concern is about actually comparing the current model to some of these earlier sequence analysis methods. The authors use a Markov model as a null model to find overrepresented sequences, but given previous work suggesting hierarchical structure in behaviour sequences (e.g. Berman et al. cited in the manuscript), a hierarchical Markov model could be a useful additional comparison.

The authors mention n-gram counting as another possible alternative. They list memory requirements and noisy behavioural data as reasons to avoid it. These are indeed potential issues, but whether they matter in practice should be established. I think the authors should compare their approach to n-gram counting for two reasons: n-gram counting has the advantage of simplicity/intelligibility and it has previously been shown to work in identifying behaviour sequences that distinguish free roaming from chemotaxis (Schwarz et al. listed above). To what extent would over-represented n-grams have identified the aversive sequence bouts they describe or the thermalling strategy of the glider?

We agree with the reviewer that *n*-gram counting is an alternative that is attractive due to its simplicity. We note that BASS in its most basic form (with no pattern or syllable noise) performs the same function as *n*-gram counting.

One major advantage of BASS is the automatic selection of *n* based on statistical significance and the capability to find sequences of length beyond what could be feasibly found using a straightforward implementation of an *n*-gram model. Indeed, the abnormal sequences found by BASS are small enough that they could have been found by *n*-gram counting as well, but this is not clear *a priori* except through the application of BASS, which excludes longer sequences.

The other advantages are the generative framework underlying BASS, which can be extended in various ways, and the inclusion of pattern noise, which can be flexibly modified depending on the system under consideration. We have included this point (see lines 469-493) and others below in a new sub-section (“Relationship with other models and extensions”) in the Discussion.

The authors also contrast their approach with compression algorithms, stating that they "do not necessarily lead to meaningful motifs; for example, the two-symbol word ab could be identified as a motif simply because a and b occur often, even if a and b occur next to each other purely by chance." This might be true in principle, but it's not clear whether it occurs in practice without a more direct comparison between methods (for example, if the sequence ab is useful in compression and is found to be used more frequently in a fish doing aversive chemotaxis compared to controls it's difficult to argue it's meaningless). Another reason to consider a direct comparison is that compression has a distinct advantage over the current approach in computational complexity. The authors discuss the challenge of scaling their approach to larger datasets. Several algorithms in the Nevill-Manning and Witten compression paper the authors cite have linear-time implementations which makes them easy to scale to large behavioural datasets. Knowing which approach to choose for large data sets would be easier if there were a better analysis of the actual advantages and disadvantages of BASS compared to compression.

The key issue with the compression algorithms is that they find motifs that may not be an intrinsic property of the data generation process, i.e., there is no generative model assumed. This means that the same algorithm applied on two datasets with the same data generating process can give very different results. To illustrate this point, we applied the Sequitur (Nevill-Manning) algorithm on zebrafish larvae data and showed the inconsistency of the motifs found on two halves of the exploratory dataset. This inconsistency was also reproduced on a simple dataset of sequential coin tosses. This point is expanded further in lines 494-513 as part of the new section (“Relationship with other models and extensions”).

In summary, the authors emphasise the advantages of their method (lines 58-61) and the disadvantages of other methods but don't do a comparison on actual data. Of their listed advantages, 1 and 3 are shared by n-gram counting and possibly 2, although it would depend on the size and nature of the data. Compression algorithms share all three advantages and have better scaling properties than BASS.

I would recommend the following:
1) (at minimum) Include a more complete discussion of the advantages of disadvantages of BASS and other methods.

Please see the new section “Relationship with other models and extensions”.

2) (probably worth doing) Include a comparison between BASS and n-gram counting.

Please see comment above. We have discussed the relationship between the two in lines 469-493.

3) (possibly worth doing) Include a comparison between BASS and an HHMM.

We have discussed the relationship between BASS and HHMM in more detail (see lines 514-525). The key disadvantages of HHMM are that the exact implementation has complexity L^3, where L is the size of the dataset and inference requires fitting O(DK^2) parameters, where D is the dictionary size and K is the alphabet size.

4) (possibly worth doing) Include a comparison between BASS and one of the compression algorithms.

Please see comment above. We have discussed the relationship between the two in lines 494-513.

Reviewer #2:

 In this paper, the authors propose a novel method for analyzing locomotion data under various environmental conditions without accessing stimulus information; they extract sequences (motifs) that rarely occur in the noisy behavioural data obtained from freely-moving animals. They investigate a lexical, hierarchical model of behaviour whose two layers map these motif templates to a latent state space which then underlies the noisy observed action outputs. Further they developed an unsupervised inference algorithm called BASS to identify such motif templates in a given dataset, find their corresponding probabilities and determine how they may underpin the observed behaviour. Finally the authors confront their generative model and inference method using synthetic data, experimental observations of zebrafish (normal and novel chemotaxis assay) and simulated data of soaring gliders. In these sections they also quantify the non-markovian nature of their derived sequences. Additionally, in case of the zebrafish they comment on the implications of their differing motif dictionaries.

The paper is very interesting and presents new innovative and interesting results. Therefore, to my opinion, the manuscript clearly deserves publication in PLoS Computational Biology. However, I have a few detailed remarks, which I list below.

We thank the reviewer for the kind assessment and comments.

1. It is not clear from the outset what conserved means. My understanding is this means behaviour conserved across different environments (such as circle and square swim areas for the zebrafish) and across genetic variations. Could the authors please clarify this in the introduction?

The conserved motifs refer to sequences of locomotor bouts that recur in the behavioral time series. We understand the reviewer’s confusion: to avoid ambiguities in the definition of conserved, we swapped ‘conserved’ for ‘recurring’ throughout the revised manuscript.

2. Please check that $p_d$ is referred to as probability of deletion (not insertion) in the manuscript (for eg. Caption of fig. 2, line 254).

Done.

3. In fig. 2 based on the text I am inferring that 2(e) also uses the same parameters as (b) and (c). Please clarify that this is the case since the parameter values differ in the different subplots

We have clarified this in the caption for Figure 2.

Additionally, I would interested in seeing a similar set of subplots for $epsilon_p \neq 0$ as a benchmark for future experimental analysis that is presented in the paper -- need to do

We performed further simulations, which can be found in a new figure (Figure S1) of the revised manuscript.

4. To assist the reader, I would suggest the authors add a sentence after 211 / in the figure caption stating why optimality is not 100% but ~ 90%.

Done, please see lines 222-223.

5. The authors often state that the motifs are rare but then also include that motifs in the case of the moving zebrafish covered 78% of all bouts per fish. I believe it would help clarify the seeming dichotomy if the authors could include a column in tables 1 and 2 stating the fraction of a dataset covered by a motif.

We have included an extra column in Tables 1 and 2 showing the coverage, i.e., the fraction of the dataset tiled by the corresponding motif.

6. Table S1 : change 50 to 25.

Done.

**Additional changes:**

- For clarity, we note that locomotor episodes are equivalent to bouts in the introduction (line 20).
- We observed that the abnormal fTff (which we noted previously as a false positive) indeed did not appear when the entire analysis pipeline including the clustering was re-run. We have included a comment to this effect (lines 384-388).
- Line 699 had an erroneous phrase (“whose components sum to one”) which has been deleted in the un-highlighted submission of the revised manuscript. The components do not sum to one as these are not the mixture weight vectors but rather likelihoods. The change does not affect the rest of the methods or results.
